# Patient perceptions about anesthesia and anesthesiologists before and after surgical procedures

**DOI:** 10.1590/S1516-31802011000400005

**Published:** 2011-05-05

**Authors:** Fernanda Leite, Leopoldo Muniz da Silva, Sckarlet Ernandes Biancolin, Adriano Dias, Yara Marcondes Machado Castiglia

**Affiliations:** I Postgraduate Student of Anesthesiology, Faculdade de Medicina de Botucatu (FMB), Universidade Estadual Paulista (Unesp), Botucatu, São Paulo, Brazil.; II Undergraduate Student, Faculdade de Medicina de Botucatu (FMB), Universidade Estadual Paulista (Unesp), Botucatu, São Paulo, Brazil.; III PhD. Epidemiologist and Assistant Professor, Faculdade de Medicina de Botucatu (FMB), Universidade Estadual Paulista (Unesp), Botucatu, São Paulo, Brazil.; V MD, PhD. Titular Professor, Department of Anesthesiology, Faculdade de Medicina de Botucatu (FMB), Universidade Estadual Paulista (Unesp), Botucatu, São Paulo, Brazil.

**Keywords:** Anesthesia, Ethics, General surgery, Human resources, Perioperative care, Anestesia, Ética, Cirurgia geral, Recursos humanos, Assistência perioperatória

## Abstract

**CONTEXT AND OBJECTIVE:**

Anesthesiologist-patient relationships are established preoperatively and intraoperatively. These are opportunities for providing correct information about anesthesia/anesthesiologists, thereby improving outcomes. The aim here was to evaluate patients’ perceptions about anesthesiologists before anesthesia and to identify whether the anesthetic care would change such perceptions.

**DESIGN AND SETTING:**

Prospective cross-sectional study using data obtained in 2007-2008, at a tertiary university hospital.

**METHODS:**

518 patients aged 16 years or over were interviewed before and after anesthesia exposure. A questionnaire was used to determine patient characteristics and perceptions of anesthesia/anesthesiologists.

**RESULTS:**

The patients were 16-89 years of age and 59.8% had attended elementary school. 79.1% said that anesthesiologists were specialized physicians. Anesthesiologists’ roles were associated with loss of consciousness (35.5% pre-anesthesia; 43.5% post-anesthesia), pain relief (29.7% pre-anesthesia, 31.7% post-anesthesia), vital sign monitoring (17.6% pre-anesthesia, 35% post-anesthesia; P < 0.05); and drug administration (10.8% pre-anesthesia, 43.9% post-anesthesia; P < 0.05). The level of confidence in the physician was rated high (82.2% and 89.8% pre- and post-anesthesia, respectively; P < 0.05) or intermediate (5.8% and 6.6% pre- and post-anesthesia, respectively; P < 0.05). The care provided by anesthesiologists was classified as: elucidating (52.8%), encouraging (52.6%), neutral (10.2%) and careless (0.8%).

**CONCLUSION:**

Patients’ perceptions of anesthesiologists’ roles were fairly good, but improvements in this relationship still need to be pursued, to achieve better outcomes. Anesthetic care was important in providing information, confidence and reassurance among patients, regarding their perceptions. Anesthesiologists should not miss opportunities to provide excellent professional care for patients, thereby improving anesthesia outcomes and their image.

## RESUMO

**CONTEXTO E OBJETIVO:**

A relação anestesiologista-paciente é estabelecida no pré-operatório e intraoperatório, oportunidades que proporcionam informações corretas sobre anestesia/anestesiologista, melhorando seus resultados. O objetivo foi avaliar a percepção dos pacientes sobre o anestesiologista antes da anestesia e se os cuidados anestésicos poderiam mudar essa percepção.

**TIPO DE ESTUDO E LOCAL:**

Estudo prospectivo transversal com dados em 2007-2008, em hospital universitário terciário.

**MÉTODOS:**

Foram entrevistados 518 pacientes com 16 anos ou mais antes e depois da exposição à anestesia. Um questionário determinou as características do paciente e da percepção da anestesia/anestesiologista.

**RESULTADOS:**

Os pacientes tinham entre 16 e 89 anos e 59,8% possuíam ensino fundamental, 79,1% responderam que o anestesiologista é um médico especializado. O papel do anestesiologista foi associado à perda de consciência (35,5% pré-anestesia, 43,5% pós-anestesia), alívio de dor (29,7% pré-anestesia, 31,7% pós-anestesia), monitorização dos sinais vitais (17,6% pré-anestesia, 35% pós-anestesia; P < 0,05), e administração de medicamentos (10,8% pré-anestesia, 43,9% pós-anestesia; P < 0,05). O nível de confiança no médico foi considerado alto (82,2% e 89,8% no pré e pós-anestesia, respectivamente, P < 0,05) ou intermediário (5,8% e 6,6% no pré e pós-anestesia, respectivamente, P < 0,05). A assistência prestada pelo anestesiologista foi classificada como: elucidativa (52,8%), encorajadora (52,6%), indiferente (10,2%) e displicente (0,8%).

**CONCLUSÃO:**

A percepção sobre o papel do anestesiologista foi satisfatória, mas as melhorias nessa relação ainda são necessárias para se conseguirem melhores resultados. O atendimento anestésico foi importante para a informação, confiança e segurança do paciente sobre essa percepção. O anestesiologista não deve perder oportunidades de exercer excelentes cuidados profissionais para os pacientes, melhorando os resultados de sua anestesia e sua imagem.

## INTRODUCTION

For all patients, and also for the physician's sake, every medical approach must cause more good than harm, and this is the basis for all medical practice and ethical relationships. Thus, every time a decision is made, medical practice should require balancing of medical approaches against patients’ problems, in order to prevent errors.^1^

The anesthesiologist-patient relationship is established during the preoperative visit, which is an occasion at which physicians and patients examine each other. The preoperative visit is the best, if not the only opportunity to provide patients with correct information about the anesthetic procedure. Studies on knowledge, attitudes and concerns regarding anesthesia, as well as regarding anesthesiologists’ image have suggested that talking to patients during the preoperative visit can enhance their confidence in the anesthetic procedure.^2^ Moreover, the benefits from recent advances that have reduced the risks rather than just the hazards of anesthetic practice should be actively promoted among the population,^3^ in order to improve the strategies for better anesthesia-surgery outcomes.

In 1993, a preoperative survey among the patients attending our teaching hospital showed that only 58% of these individuals knew that anesthesiologists were specialized medical physicians, while 31.8% associated them with pain relief and 24.5% with loss of consciousness. The confidence level was high among 76.5% of the patients, and 92.8% of them said that they would rather not choose their own anesthesiologist.^4^ This scenario could and should be improved. Because these results were obtained 15 years ago, the present study aimed to assess patients’ perceptions regarding anesthesia and anesthesiologists, and whether the interaction between physicians and patients during the preoperative visit and during the anesthetic procedure (i.e. the humanization process) might change patients’ previous views of anesthesia and anesthesiologists.

## OBJECTIVE

The aim of this study was to evaluate the patients’ knowledge about the professional condition of the anesthesiologist on two occasions: preoperative and postoperative; and to identify whether anesthetic care is a key factor for changes in this perception.

## METHODS

This cross-sectional before-and-after study was based on opinions and informations provided before and after anesthesia by individuals who had been hospitalized in the Teaching Hospital of the Faculdade de Medicina de Botucatu (FMB) to undergo surgery under anesthesia administered by the Department of Anesthesiology of the FMB.

The study population consisted of a 12-month consecutive sample of Portuguese-speaking surgical patients aged 16 years or over. Patients with altered states of consciousness or impairment of expression/comprehension, as well as those who did not undergo anesthesia/surgery (due to cancellation or postponement) or whose discharge occurred before a post-anesthesia interview could be conducted, were excluded. The same patient gave responses to both questionnaires (pre and post-anesthesia).

The preoperative data were collected 16 hours before the operation, and the post-anesthesia data, 24 hours after the end of the surgical procedures. The sample size for this study was estimated from the results of a previous investigation.^4^ Thus, at least 455 patients needed to be evaluated to reach a statistical power of 90% and confidence interval of 95%.^5^ We did not create *a priori* subgroups. There were no losses from the sample.

The chi-square test was used to compare proportions and to investigate associations among variables. Age was recorded as mean and standard deviation. The significance level was set at 5%.^6^

Informed written consent was obtained from all patients and the study was approved by the Research Ethics Committee of FMB, Universidade Estadual Paulista (Unesp).

### Data gathering instrument

Sixteen hours before anesthesia, the patients were presented with a questionnaire including structured questions to identify patient characteristics (age, gender, race, marital status, education level, birthplace, provenance, surgical service of origin, occupation and history of previous anesthesia exposure) and perceptions of anesthesiologists/anesthesia (objective knowledge of the role and training of anesthesiologists, confidence in the professional, whether they would rather choose the anesthesiologist if possible, preferences regarding anesthetic procedures, and concerns about the anesthetic procedure). Twenty-four hours after the end of anesthesia, the patients were once again interviewed in order to determine whether their perceptions of anesthesiologists/anesthesia had changed. The post-anesthesia interview also included questions regarding the administration of pre-anesthetic drugs, patients’ recall of the anesthesiologist in the operating room and patients’ satisfaction with the overall care provided by the anesthesiologist.

The data were gathered between May 2007 and May 2008. The pre-anesthesia and post-anesthesia interviews were conducted by trained personnel: two of the authors (FL and SEB), with collaboration from residents in the first, second and third years of residency in FMB Department of Anesthesiology. Both interviews were conducted at the bedside.

## RESULTS

During the study period, 518 patients (55.6% females) aged between 16 and 89 years (46.9 ± 15.6 years) were interviewed. Of these, 81.9% were white and 18.1% were non-white. Regarding marital status, 63.7% were married, 20.5% single and 15.8% widowed, separated or divorced.

It was found that 34.5% of the patients were rural or urban employed workers, 22.2% were homemakers (all women), 19.1% were retired, 15.6% were self-employed workers, 3.5% were students, 3.5% were unemployed and 1.4% were unable to work.

Only 18.5% of the patients were natives of Botucatu, 65.8% were born elsewhere in the interior of the state of São Paulo, and 15.7% were born in other Brazilian states. Regarding their current place of residence, 33% were living in Botucatu, 65.5% were living elsewhere in the interior of the state of São Paulo and 1.5% were living in other states.

Concerning education, 3.5% were illiterate, 59.8% had only attained an elementary level (either completed or incomplete), 25.5% had attained high school level (either completed or incomplete) and 11.2% had attained higher education level (either completed or incomplete).

The variables of gender, race, marital status, age, place of residence, birthplace, educational level, clinic of origin and occupation were heterogeneously distributed. The sociodemographic data on the study population are shown in Table 1.

**Table 1.** Sociodemographic data on 518 patients interviewed before and after anesthesia at Botucatu Medical School Hospital between May 2007 and May 2008

Variables	n = 518	%
Gender		
Female	288	55.6
Male	230	44.4
Race		
White	424	81.9
Brown	56	10.8
Black	38	7.3
Marital status		
Married	330	63.7
Single	106	20.5
Widowed	48	9.3
Other	34	6.5
Birthplace		
Botucatu	96	18.5
São Paulo interior	341	65.8
Other	81	15.7
Place of residence		
Botucatu	171	33.0
São Paulo interior	339	65.5
Other	8	1.5
Educational level		
Elementary	310	59.8
High school	132	25.5
Higher	58	11.2
Illiterate	18	3.5
Occupation		
Employed (urban/rural)	179	34.5
Homemaker	115	22.2
Retired	99	19.1
Self-employed	81	15.6
Student	18	3.5
Unemployed	18	3.5
Disabled	8	1.4

The operations performed included gastric (19.1%), gynecological (18%), orthopedic (15.3%), urological (13.5%), vascular (6.2%), neurosurgical (6.0%), otorhinolaryngological (5.8%), cosmetic (5.6%), ophthalmological (5.0%), chest (4.2%) and cardiac (1.3%) procedures. Seventy-six percent of the patients had previously been anesthetized.

During the pre-anesthesia assessment, 35.5% of the patients associated the role of anesthesiologists with loss of consciousness and 29.7% with pain relief; 19.1% of them were unsure about anesthesiologists’ role; 10.8% thought that anesthesiologists were mainly responsible for drug administration; and 17.6% believed they were in charge of monitoring and taking care of patients’ vital signs.

After anesthesia, 43.9% thought that the major function of anesthesiologists was to administer drugs; 43.5% associated their role with loss of consciousness; 35% believed that anesthesiologists had monitored and taken care of their vital signs; 31.7% thought that they had been responsible for pain relief; and 23.2% of the respondents were still unsure about the anesthesiologist's role (P = 0.03).

In response to the question "what are anesthesiologists?", which was asked during the pre-anesthesia visit, 79.1% of the patients answered that they were specialized physicians, 7.3% thought that they were unspecialized non-surgical physicians, 3.5% that they were surgeons, 1.0% that they were nurses, 1.0% that they were nursing auxiliaries, and 0.6% that they were surgical technicians. Furthermore, 7.5% thought that anesthesiologists had roles other than those mentioned above. At the post-anesthesia assessment, the patients showed that they thought anesthesiologists were: specialized physicians (87.4%), unspecialized physicians (3.9%), surgeons (3.9%), nurses (1.4%), nursing auxiliaries, (1.6%) or surgical technicians (0.2%), or that their role was other than those mentioned above (1.6%) (P = 0.04). The percentage of correct answers tended to be smaller among the patients of lower educational level.

The pre-anesthesia level of confidence in the physician was rated as high by most patients (82.2%), intermediate by 5.8%, and low by 1.2% of the respondents. However, 10.8% of the patients admitted not having thought about it. After anesthesia, the confidence level was rated high by most (89.8%), intermediate by 6.6%, and low by 0.8%, while 2.9% admitted that they had not thought about it even after anesthesia (P = 0.03). Educational level was not found to be correlated with the difference between pre and post-anesthesia answers.

Table 2 shows the overall results relating to patients’ views of anesthesiologists.

**Table 2.** Overall results relating to patients’ views of anesthesiologists, as reported by 518 individuals interviewed before and after anesthesia at Botucatu Medical School Hospital between May 2007 and May 2008 (n = 518)

Variables	Preoperative (%)	Postoperative (%)
Role		
Loss of consciousness	35.5	43.5
Pain relief	29.7	31.7
Unsure	19.1	23.2
Drug administration	10.8	43.9
Monitoring of vital signs	17.6	35.0
(χ^2^ = 69.5541; P = 0.0001)		
What are anesthesiologists?		
Specialized physicians	79.1	87.4
Physicians	7.3	3.9
Surgeons	3.5	3.9
Nurses	1.0	1.4
Nursing auxiliaries	1.0	1.6
Technicians	0.6	0.2
Others	7.5	1.6
(χ^2^ = 29.64; P = 0.0001)		
Confidence level		
High	82.2	89.8
Intermediate	5.8	6.6
Low	1.2	0.8
Did not think about it	10.8	2.9
(χ^2^ = 26.03; P = 0.0001)		
Concern		
Yes	29.3	7.1
No	70.7	38.5
No answer		54.4
(χ^2^ = 15.93; P = 0.001)		

When the patients were asked whether they would rather choose the anesthesiologist if possible, the majority (92.1%) said that they would not do so, for the following reasons: they did not know any anesthesiologist (41.3%), the surgeon should make the choice (23.9%), they did not feel qualified to do it (17%), and they were not interested in making the choice (11.2%). Only 7.7% of the respondents said that they would like to choose their own anesthesiologist, because they thought they had the right to do so (6.2%) or knew an anesthesiologist to choose (2.3%).

Regarding the type of anesthesia, 41.1% showed no preference, while 40.9% would choose general anesthesia, 11% mentioned spinal anesthesia, 2.3% epidural anesthesia, 6% local anesthesia and 0.4% limb blockade.

Out of all the patients interviewed, 29.3% expressed preoperative concerns. This percentage dropped to 7.1% (P = 0.02) after anesthesia exposure. The percentage of responses mentioning both preoperative and postoperative concerns was similar among patients of all levels of education.

During the pre-surgery period, 53.7% of the patients were given no pre-anesthetic medication; 43.2% received midazolam and 3.1% diazepam.

Most patients (77%) remembered seeing the anesthesiologist in the operating room. The care provided by the anesthesiologist was considered elucidating by 52.8%, encouraging by 52.6%, neutral by 10.2% and careless by 0.8%.

## DISCUSSION

This investigation primarily focused on identifying our typical patient profile. This was found to be an individual aged between 40 and 50 years, female, white, with a rural or urban job and elementary education level (either completed or incomplete), married, born and living in the state of São Paulo, exposed to anesthesia at least once, and subjected to gastroenterological, gynecological or orthopedic surgical procedures. Taking into account how quickly news and ideas can be spread through the media today, information on the anesthetic procedure and the actual role of anesthesiologists could relatively easily reach such individuals, provided that investment policies were developed to disseminate this kind of information.

Visit length influences patient satisfaction with the physician-patient relationship.^7^ Unfortunately, because of the large number of patients who have to be seen, anesthesiologists do not always give this point its deserved attention. Moreover, the strong emotional context, the potential drug cognitive effects and the short time interval between the preoperative visit and the anesthetic procedure, as well as the brief duration of the contacts make it even harder to assess patient satisfaction with anesthesia services.

In contrast with the findings reported by Lopes et al.,^4^ the present results show that, preoperatively, the role of anesthesiologists was mostly associated with loss of consciousness followed by pain relief. The rate of knowledge that anesthesiologists are specialized physicians was 20% greater in our study. The frequency of this response significantly increased after anesthesia. The dissemination of information on the actual role of anesthesiologists improved. The introduction made by the anesthesiologist during the preoperative visit or the questionnaire itself may have influenced patients’ judgment after anesthesia. Nonetheless, the knowledge of anesthesiology as a medical specialty identified in this study might be explained by the fact that the study population was aware that our hospital is a university hospital where newly graduated physicians attend specialization programs.

The patients had considerable recall of the anesthesiologists’ presence in the operating room, and the patients’ perception of their role went up by 8% after anesthesia, thus showing the importance of the anesthesiologist-patient relationship. The percentage of responses associating anesthesiologists with drug administration and vital sign monitoring substantially increased after anesthesia, whereas the rates of association with loss of consciousness and pain relief, or even uncertainty about anesthesiologists’ role were similar before and after anesthesia. It should be noted, however, that a number of patients had received pre-anesthetic medications, such as midazolam, which induce amnesia in most cases. Furthermore, a considerable number of the respondents had a low educational level, which might have prevented them from perceiving exactly what went on the surgical center.

The physician-patient relationship and its outcomes can be strengthened by providing patients with clear guidance and using words and explanations that are appropriate to patients’ levels of comprehension, in order to avoid underestimation of anesthesiologists’ role caused by situations that are unfavorable to perception *per se*. As a result, confidence in the physician will hopefully increase, since anesthetic-surgical stress and concerns will be reduced. Historically, the American Society of Anesthesiologists (ASA) has supported a strong campaign to improve the public perception and understanding of anesthesiologists’ role.^8^ To achieve this mark, open relationships should be built to improve the physician-patient relationship.

Studies conducted in a Caribbean country and in Ireland have also shown that patients still have a very limited view of anesthesiologists’ role.^9,10^ Although 59.0% knew that anesthesiologists were physicians, a tenth of the respondents did not know what anesthesiologists were.^9^ Moreover, in agreement with our findings, the educational level of the respondent was significantly associated with this response.

Another point that might reflect the characteristics of the service is the high level of confidence in anesthesiologists expressed by most respondents. Our hospital is known to be a university hospital where patients are passively introduced into the system, such that they are unable to choose their specialized physician, date of admission, time of surgery, etc. The high level of confidence in the physician reported preoperatively increased by almost 8% after surgery. Nonetheless, the average level of confidence showed an increase postoperatively. Individuals facing the unknown normally create fantasies and they emotionally react to them by stressing good or bad responses.^11^ Thus, it is important for patients to feel integrated (with the surgical center environment and caregivers). In addition, the way in which institutions and physicians receive patients and meet their demands determines how patients will take part in the therapeutic process.^11^ Moreover, if patients know about the anesthetic-surgical process and also have contact with anesthesiologists both during the preoperative visit and during the anesthetic procedure, a large number of preoperative concerns may disappear. This is a positive point for establishing an effective physician-patient relationship.

Finally, it should be noted that for ethical reasons mainly relating to patient safety, it was not possible to use a control group without a pre-anesthetic visit in the present study, although such a control group would definitely have certified the reliability of the results found.

Thus, anesthesiologists should not miss any opportunities to get professionally involved outside the operating room, whether in preoperative assessments, antalgic therapy, obstetric care, intensive care services or as active members of the hospital medical staff. In the final analysis, they can improve their image in the eyes of the population that they assist. The objective of further improving patients’ perceptions of anesthesiologists’ roles in the operating room, might be achieved through institutional videos in ambulatory surgical services. These might reduce anxiety and increase comprehension of the function of anesthesia during surgery. These issues could also be better explored during the bedside interview. Appropriate perception of anesthesiologists’ role could be improved at the time of patients’ pre-anesthetic ambulatory evaluation.

## CONCLUSION

In conclusion, the patients’ perceptions of anesthesiologists’ roles were fairly good, but improvements in this relationship still need to be pursued, in order to achieve better outcomes. Anesthetic care was important in providing information, confidence and reassurance among patients, regarding their perceptions.
